# The metabolic flux regulation of *Klebsiella pneumoniae* based on quorum sensing system

**DOI:** 10.1038/srep38725

**Published:** 2016-12-07

**Authors:** Shujing Sun, Haiyang Zhang, Shuyi Lu, Chunfen Lai, Huijun Liu, Hu Zhu

**Affiliations:** 1 College of Life Sciences, Fujian Agriculture and Forestry University, Fuzhou 350002, PR China; 2 Centre for Bioengineering and Biotechnology, China University of Petroleum (East China), Qingdao 266580, PR China; 3 College of Plant Protection, Fujian Agriculture and Forestry University, Fuzhou 350002, PR China

## Abstract

Quorum-sensing (QS) systems exist universally in bacteria to regulate multiple biological functions. *Klebsiella pneumoniae*, an industrially important bacterium that produces bio-based chemicals such as 2,3-butanediol and acetoin, can secrete a furanosyl borate diester (AI-2) as the signalling molecule mediating a QS system, which plays a key regulatory role in the biosynthesis of secondary metabolites. In this study, the molecular regulation and metabolic functions of a QS system in *K. pneumoniae* were investigated. The results showed that after the disruption of AI-2-mediated QS by the knockout of *luxS*, the production of acetoin, ethanol and acetic acid were relatively lower in the *K. pneumoniae* mutant than in the wild type bacteria. However, 2,3-butanediol production was increased by 23.8% and reached 54.93 g/L. The observed enhancement may be attributed to the improvement of the catalytic activity of 2,3-butanediol dehydrogenase (BDH) in transforming acetoin to 2,3-butanediol. This possibility is consistent with the RT-PCR-verified increase in the transcriptional level of *budC*, which encodes BDH. These results also demonstrated that the physiological metabolism of *K. pneumoniae* was adversely affected by a QS system. This effect was reversed through the addition of synthetic AI-2. This study provides the basis for a QS-modulated metabolic engineering study of *K. pneumoniae*.

A communication system among bacteria was first discovered in the 1970s while studying the luminescence mechanism of *Photobacterium fischeri*^1^. This phenomenon was observed in the luminescence organs of marine organisms and was named quorum sensing (QS)^2^. In bacterial cells, QS consists of small, diffusible signalling molecules (autoinducers) capable of sensing the density of bacterial cells and subsequently initiating the coordinated expression of several key genes throughout the entire bacterial community when the autoinducer concentration exceeds a critical threshold in the cells^3^,^4^. Recent research has led to the discovery of some new autoinducers in bacteria, illustrated how these autoinducers are recognized by cognate receptors, revealed new regulatory components embedded in canonical signalling circuits and identified novel regulatory network designs^5^. At the same time, QS is also a mechanism for bacterial adaptation to the environment. QS systems exist in multiple bacterial species^3^, and the luxR family of proteins in this system play a key role as transcription regulators^2^. With LuxI protein and signalling molecules, QS systems can modulate a variety of physiological functions, such as bioluminescence^1^, symbiosis^6^, Ti plasmid conjugative transfer^7^, biofilm formation^8^, group mobility, pathogenesis^9^, among others. Notably, the QS system in *Pseudomonas aeruginosa* involves two discrete acyl-homoserine lactone (AHL) molecules (OdDHL and BHL) that are generated and sensed by two different signalling systems (LasIR and RhlIR), which control the production of a diverse variety of virulence factors and biofilm formation, respectively^4^. Recently, the emergence and worldwide spread of antibiotic-resistant bacteria have increased the importance of finding therapeutic alternatives to compensate for the reduced effectiveness of antibiotics^10^. QS systems have also been suggested as promising targets for developing new anti-infective compounds based on the regulatory function of these systems in the pathogenesis of bacteria^11^. It is reasonable to consider that targeting the QS system would put less selective pressure on pathogens, thus avoiding the development of resistant bacteria and combating some of the most hard-to-treat infections that are resistant to powerful antibiotics^11^. Fundamental research into the QS mechanism has revealed suprising discoveries^4^. For example, the signalling molecule *N*-(3-oxododecanoyl)-l-homoserine lactone can cause inflammation and induce immunomodulatory activity^12^, which may reveal new drug targets.

Currently, the gradual exhaustion of petroleum has revived significant interest in producing bio-based bulk chemicals, including 2,3-butanediol, from biomass^13^. The demand and manufacture of 2,3-butanediol are increasing worldwide due to the extensive industrial applications of this chemical^14^. For example, 2,3-butanediol can be used as a liquid fuel additive because of its high combustion value (27.198 Jg^−1^), as an antifreeze agent due to the low freezing point of −60 °C, as a carrier for pharmaceuticals and as a precursor in the production of methyl ethyl ketone and 1,3-butadiene through dehydration. In addition, 2,3-butanediol is easily dehydrogenated into acetoin and diacetyl, which can be used in food and cosmetics similar to flavouring agents^14^,^15^. *Klebsiella pneumoniae*^16^, a Gram-negative bacterium, is an industrially important bacterium that produces bio-based chemicals, including 2,3-butanediol and acetoin^17^. *K. pneumoniae* contains AI-1 type QS quenching enzymes, AHL lactonases^18^. The AHL lactonases in prokaryotes can be categorized into 2 clusters: *AiiA* and *AttA*. The lactonase from *K. pneumoniae* belongs to the *AttA* cluster and shares only 30% homology with lactonases from other species^18^,^19^. In the submerged fermentation of *K. pneumonia*, because AHL lactonase is present, AHLs cannot reach the threshold concentration in the culture medium, cannot diffuse back into the cell and cannot be recognized by the receptor protein. Therefore, an AHL-receptor protein complex cannot form, and the AHL-mediated QS system does not work. However, *K. pneumoniae* contains *metK, pfs* and *luxS* genes, which all encode key enzymes in the synthesis of the signalling molecule AI-2, suggesting that *K. pneumoniae* uses AI-2 as the signalling molecule in a QS system to regulate group behaviours^20^,^21^. In nature, the tight regulation of AI-2 production by *K. pneumoniae* occurs primarily at the level of *luxS* transcription in the synthetic pathway of AI-2, and the maximum AI-2 activity occurs during the late exponential phase, which was determined by our laboratory^16^. Mutations in *luxS* induce an increase in the expression of two lipopolysaccharide (LPS)-synthesis-related genes, *wbbM* and *wzm*, which affect the biofilm formation of *K. pneumoniae*^21^. As such, this *LuxS*-dependent signal also plays a main role in the early stages of biofilm formation by *K. pneumoniae*^20^. Therefore, it was concluded that AI-2 acts as a regulator of biofilm formation and LPS synthesis in *K. pneumoniae*^21^. According to the primary literature, 2,3-butanediol fermentation is dependent on an AHL-dependent QS system in *Serratia* spp.^22^. Although 2,3-butanediol production by *K. pneumoniae* has also been well studied, it was not known how 2,3-butanediol was regulated by an AI-2-mediated QS system until now.

In this study, the molecular regulation and metabolic function of the QS system in *K. pneumoniae* was analysed based on previous studies of the cellular metabolic network of *K. pneumoniae* and transcriptional characteristics of the QS system. Additionally, a drift in the metabolic flux and changes in the physiological metabolic network of *K. pneumoniae* were also investigated. The results presented here will lay the foundation for elucidating the interplay between industrial microbial metabolism and QS systems.

## Results

### Regulation of the AI-2-mediated QS system in *K. pneumoniae*

**Figure 1:**
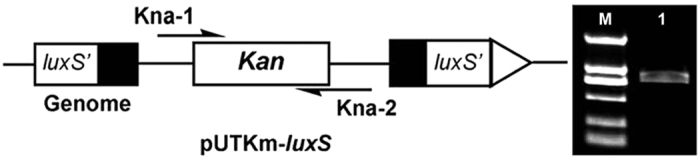
Detection of luxS mutants using PCR amplification. Left panel: Schematic diagram of the suicide plasmid insertion mutation; Right panel: Agarose gel electrophoresis of the partial fragment of the Kanamycin gene. Lane M: DNA marker D2000 (top to bottom: 2.0, 1.0, 0.75, 0.5, 0.25, 0.1 kb); Lane 1: Partial fragment (800 bp) of the Kanamycin gene.

The QS system of *K. pneumoniae* is *luxS*-dependent^16^; therefore, it is possible to regulate QS by inactivating a key gene, *luxS*, through mutagenesis. The gene product of *luxS* is involved in the synthetic pathway of the signalling molecule AI-2. Chromosomal *luxS* was inactivated in each strain using a marker-exchange strategy based on the suicide vector pUTKm as described in the Materials and Methods. The recombinant plasmid pUTKm-*luxS* was introduced into *K. pneumoniae* CICC 10018 competent cells by electrotransformation, followed by screening of the kanamycin-resistant *luxS* mutants^16^. Bacterial growth on the kanamycin plates was evident, suggesting that the *luxS* gene was knocked out. Clones that were kanamycin-resistant were verified by PCR assays of genomic DNA. An 800-bp (as expected) fragment of the kanamycin-resistance gene was PCR-amplified with primers Kna-1 and Kna-2 (Fig. 1). Additionally, the samples were also verified by commercial sequencing. The correct recombinant strain containing a kanamycin-resistance gene insertion in a chromosome was picked out (named *K. pneumoniae-6*) and was cultured in LB medium for further experiments.

### Effects of QS quenching on the metabolic flux of *K. pneumoniae*

**Figure 2:**
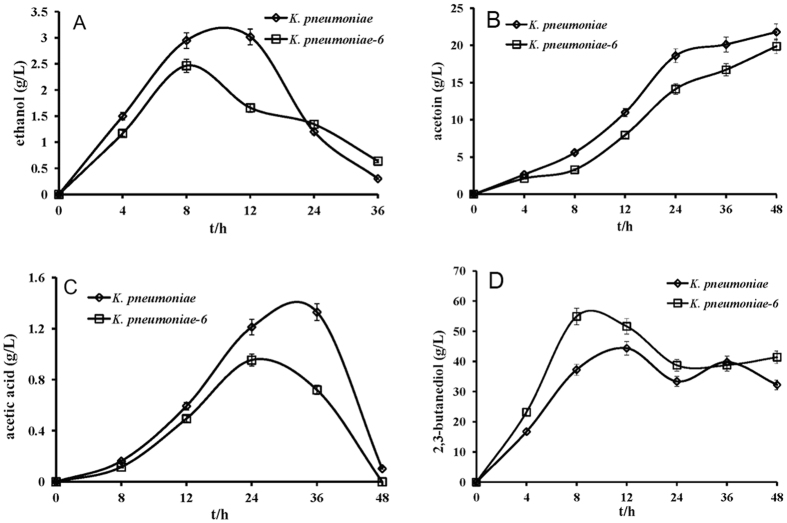
Comparison of the metabolic properties of *K. pneumoniae* (wild-type) and *K. pneumoniae-6 (luxS* mutant) in batch culture in a 5-L stirred tank bioreactor. The data are the means of three replicates. Symbols: ethanol (**A**), acetoin (**B**), acetic acid (**C**), and 2,3-butanediol (**D**).

As shown in Fig. 2, the production of 2,3-butanediol initially increased and then declined in both *K. pneumoniae* and *K. pneumoniae-6* during fermentation. The production of 2,3-butanediol in *K. pneumoniae* reached a peak at 12 h, whereas the peak production in *K. pneumoniae-6* occurred at 8 h. A possible explanation for this observation is the enhanced bioconversion to 2,3-butanediol under the catalysis of related enzymes in the metabolic flux. During this process, the production of acetoin, acetic acid and ethanol was relatively lower in *K. pneumoniae-6* than in *K. pneumoniae*, which may be attributed to the carbon flux shift because there was almost no change in the catalytic activity of related enzymes involved in the biosynthesis of these compounds (data not shown). Notably, the decreased levels of these 3 compounds in the *luxS* mutant could be restored to the levels in the parental strain in the presence of 5 μM synthetic AI-2. Although *K. pneumoniae-6* grew less well than its parent strain, the defective growth of *K. pneumoniae-6* could also be nullified by the addition of 5 μM synthetic AI-2.

### Comparison of enzymatic activity during metabolism

**Figure 3:**
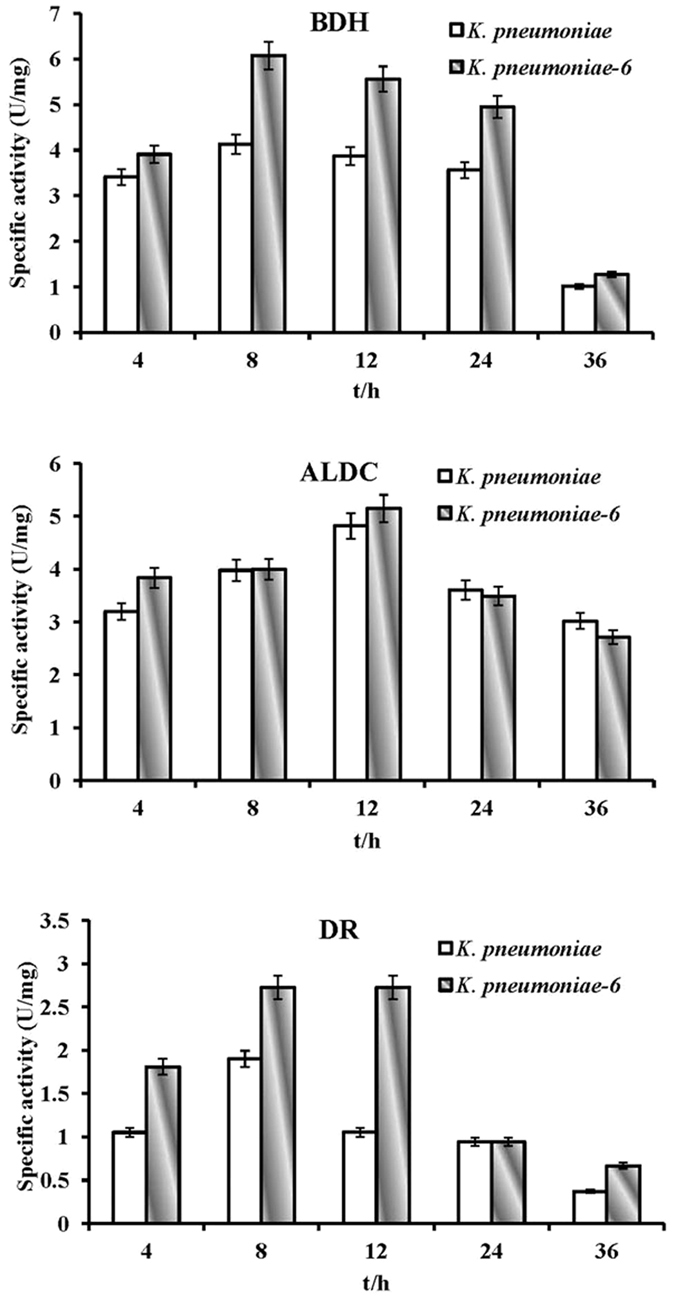
Comparison of the changes in enzymatic activity involved in the biosynthesis of metabolites produced by *K. pneumoniae-6*. The data are the means of three repeats. Symbols: 2,3-butanediol dehydrogenase (BDH), α-acetolactate decarboxylase (ALDC), and diacetyl reductase (DR).

There are two metabolic pathways in bacteria for the biosynthesis of 2,3-butanediol from α-acetolactic acid. In the first, α-acetolactic acid is converted to acetoin (the key precursor of 2,3-butanediol) by the catalysis of α-acetolactate decarboxylase (α-ALDC). Next, acetoin is converted to 2,3-butanediol through the catalysis of 2,3-butanediol dehydrogenase (BDH). In the second metabolic pathway, α-acetolactic acid is oxidized to diacetyl, and diacetyl is converted to acetoin through the catalysis of diacetyl reductase (DR). Subsequently, acetoin is converted to 2,3-butanediol through the catalysis of BDH. Therefore, the enzymatic activities of BDH, α-ALDC and DR in *K. pneumoniae* and *K. pneumoniae-6* were measured at the indicated time points after preparing a crude enzyme solution. Figure 3 shows that the activities of these 3 enzymes first increased and then declined with time in both strains. The maximal enzymatic activities of BDH, α-ALDC and DR occurred at similar times in both strains, at 8 h, 12 h and 8 h, respectively. The enzymatic activity of α-ALDC was similar in both strains, whereas the enzymatic activity of BDH and DR was relatively lower in *K. pneumoniae*. This lower DR activity level decreased the synthesis of acetoin from diacetyl and increased the accumulation of diacetyl in the fermentation broth of *K. pneumoniae*. Additionally, the activity of α-acetolactate synthase, which catalyses the biosynthesis of α-acetolactic acid from pyruvic acid, was also determined, but there was no significant difference between these two strains (data not shown). In *K. pneumoniae*, the enzymatic synthesis and metabolic control of 2,3-butanediol and acetoin were tightly regulated by QS. The knockout of *luxS* led to the destruction and functional disability of the QS system. As a result, 2,3-butanediol production was improved, and it was confirmed that this improvement was caused by a change in the expression level of enzymes (BDH and DR) involved in the synthetic pathway of 2,3-butanediol. These results suggest that the knockout of *luxS* can result in variations in enzymatic activities and further affect 2,3-butanediol biosynthesis. According to these results, a number of conclusions can be drawn. After quenching the QS system in *K. pneumoniae-6*, the activity of DR improved significantly, promoting the conversion from diacetyl to acetoin. The increased activity of BDH accelerated the biosynthesis of 2,3-butanediol using acetoin as a substrate, and therefore, the production of 2,3-butanediol was greatly enhanced.

### Analysis of budC transcription

**Figure 4:**
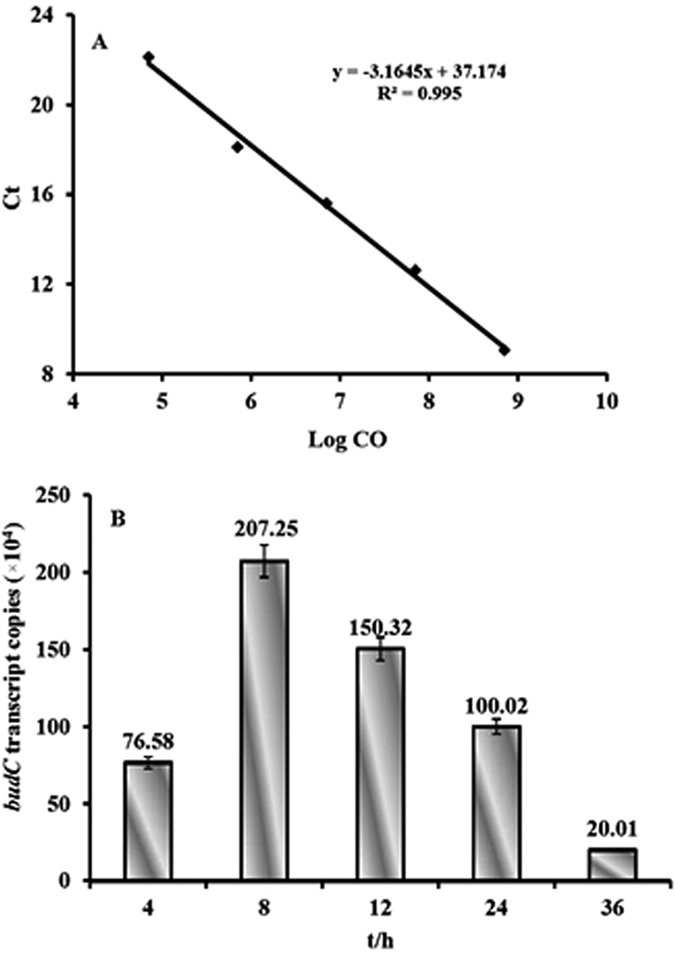
Transcription profiles of *budC* determined by RT-PCR with the primers F-budC and R-budC. The copies were calculated from the equation of the straight line in (**A**), which is the RT-PCR standard curve for the *budC* gene. The logarithms (base 10) of different concentrations are plotted against crossing points in (**B**). All the experiments were performed in triplicate.

The gene *budC* encodes BDH, which catalyses the reaction of acetoin to 2,3-butanediol. As depicted in Fig. 4, the *budC* gene was constitutively expressed at the tested time points. Additionally, the number of *budC* transcripts first increased and then decreased in *K. pneumoniae-6*, and the maximal transcription levels occurred at 8 h. This result may explain why the maximal enzymatic activity of BDH and the maximal 2,3-butanediol production took place at 8 h. During the exponential growth phase in *K. pneumoniae-6*, the *budC* gene expression increased dramatically to a maximal value. However, *budC* expression decreased upon entry into the stationary growth phase. Maximal 2,3-butanediol production and *budC* expression occurred at the same time point (8 h), which suggests that the difference in 2,3-butanediol production by *K. pneumoniae* and *K. pneumoniae-6* is due to changes at the transcriptional level of the *budC* gene during the growth of these two bacteria.

## Discussion

The chemical butanol is a four-carbon diol that has wide industrial applications for the manufacture of bulk chemicals^23^. In general, butanol is produced from carbohydrates in submerged fermentation by *Klebsiella* spp.^24^,^25^,^26^,^27^, *Enterobacter* spp.^28^,^29^,^30^,^31^, *Bacillus* spp.^32^,^33^,^34^, and *Clostridia* spp.^35^,^36^,^37^,^38^, among others. Since butanol-producing strains were initially screened from natural environments, the yield, titre and productivity regarding butanol were frequently low, and these strains could not be used in industrial production. Therefore, the current focus is on finding new strains from natural reservoirs and constructing or modifying strains through mutagenesis, evolutionary engineering and metabolic engineering strategies to improve their production performance and compliance^23^.

*K. pneumoniae* and *Klebsiella oxytoca* are important bio-based chemical-producing bacteria and can ferment glucose primarily to 2,3-butanediol as a major fermentation end-product with relatively small amounts of acetoin, ethanol and some acids under micro-aerobic conditions because a significant amount of pyruvate from glycolysis is channelled into the butanediol pathway, through which pyruvate is transformed into butanediol by the catalysis of acetolactate synthase, α-ALDC and BDH in an orderly fashion^17^,^27^. To improve 2,3-butanediol production by *Klebsiella* spp., *strain* modification by metabolic engineering has attracted a great deal of attention around the world^17^,^24^,^25^,^26^,^27^. For example, Rathnasingh *et al*. improved the production of 2,3-butanediol in *K. pneumoniae* by knocking out the genes (*ldhA, adhE and pta-ackA*) involved in the formation of lactic acid, ethanol and acetic acid; the 2,3-butanediol production thus reached 91 g/L with a yield of 0.45 g per g glucose in batch fermentation^27^. Guo *et al*. constructed *K. pneumoniae* mutants that overexpressed α-ALS, α-ALDC, and AR to improve 2,3-butanediol production. The results revealed that 2,3-butanediol production by the recombinant *K. pneumoniae* strain (KG-rs) that overexpressed both ALS and AR was 12% higher than that of the parental strain^17^. Cho *et al*. reported that 2,3-butanediol production reached 115.0 g/L in fed-batch fermentation with pure glycerol with the construction of a double mutant (*pduC, ldhA) K. oxytoca* strain to reduce the formation of 1,3-propanediol and lactic acid^24^. This group also discovered that if acetoin reductase was overexpressed in *K. oxytoca*, acetoin accumulation was significantly reduced, and the highest titre of 2,3-butanediol (142.5 g/L) was achieved^25^.

Currently, metabolic engineering strategies at the gene level are commonly used by many scientists to reform certain special production strains. Additionally, there has been growing interest in developing novel metabolic engineering strategies at the cellular level based on the QS system in bacteria^39^,^40^. For example, in bacteria of the *Serratia* genera, 2,3-butanediol fermentation has been shown to be affected by an *spl*I-dependent QS system, and the knockout of the *spl*I gene caused a shift towards enhanced acid production. Of course, the biosynthesis of 2,3-butanediol is not completely shut off by eliminating QS. At the same time, QS also controls the production of extracellular enzymes, including chitinase, nuclease, and protease^13^,^22^. In *Vibrio cholera,* the production of 2,3-butanediol is regulated by multiple QS systems via the transcriptional activator AphA. Two QS systems use a CAI-1 autoinducer and AI-2 as signalling molecules, respectively, and act in parallel to trigger a phosphorelay circuit^41^. In *Aeromonas hydrophila*, 2,3-butanediol fermentation is regulated by AHL-mediated QS because the disruption of QS by the knockout of *ahyI*, synthesizing C_4_-HSL, results in medium acidification and blocks the metabolic switch to 2,3-butanediol synthesis^13^. Furthermore, the inactivation of the regulatory protein AhyR in QS also suppressed 2,3-butanediol fermentation. Although 2,3-butanediol production by *K. pneumoniae* has been well studied^26^,^27^, the data in this report represent the first identification of a QS system regulating 2,3-butanediol production. In this study, the effects of QS on 2,3-butanediol formation in *K. pneumoniae* were analysed in great detail. *K. pneumoniae* contains AI-1 type QS quenching enzymes, including AHL lactonase, and possesses *pfs* and *luxS* orthologues in its genome; therefore, it can be inferred that AI-2 is the signalling molecule mediating the QS system^40^. In the biosynthesis of AI-2, *luxS* and *pfs* encode a 5′-methylthioadenosine/S-adenosylhomocysteine nucleosidase and form an operon that utilizes SAH or MTA as a substrate for AI-2 production^16^. Furthermore, the tight regulation of AI-2 production is largely at the level of *luxS* transcription. Therefore, the molecular regulation of the QS system in *K. pneumoniae* was established by constructing a *luxS* mutant. Next, changes in the metabolites between *K. pneumoniae-6 (luxS* mutant) and *K. pneumoniae* (parental strain) were compared through shaking fermentation with glucose. The results indicated that after quenching the QS system, the production of acetoin, ethanol and acetic acid was relatively lower in *K. pneumoniae-6*, but the 2,3-butanediol production was increased by 23.8% and reached a maximum level of 54.93 g/L. This increase suggested that the activation of the 2,3-butanediol pathway is not QS regulated, which is different from *Serratia* spp.^22^. The precise mechanism of regulation has yet to be elucidated. This improvement effect was reversed through the addition of 5 μM synthetic AI-2. At the same time, enzymatic activity analyses revealed that there was no significant difference in α-ALDC activity between these two strains, whereas the enzymatic activities of BDH and DR were relatively lower in *K. pneumoniae*, in accordance with the transcriptional analysis of *budC.* These findings should be useful for improving 2,3-butanediol production via QS-based metabolic engineering. Furthermore, this study provides a solid basis for investigating the link between QS and bacterial physiology.

Although the development of butanol-producing microorganisms and fermentation processes has seen remarkable progress, the recovery of butanol from the fermentation broth still remains a challenging problem under industrial production conditions^23^. To develop commercially applicable techniques for the recovery of 2,3-butanediol, numerous methods have been proposed^42^, *including* ion exchange, electrodialysis, membrane filtration, pervaporation, reactive extraction and liquid-liquid extraction^43^,^44^. However, these methods have inherent drawbacks that must be overcome, such as generating a large amount of wastewater for resin regeneration, requiring expensive membranes, increasing filtration steps and decreasing the recovery yield of 2,3-butanediol^42^. In recent years, many scientists have been developing *in situ* product recovery techniques, especially for the low concentration of 2,3-butanediol in fermentation broth. Jeon *et al*. established a four-step recovery process through alcohol precipitation and vacuum distillation, and a recovery yield of 76.2% and a purity of 96.1% were obtained from fermentation broth containing approximately 90 g/L of 2,3-butanediol produced by the *ldhA*-deficient *K. pneumoniae*^42^. Xue *et al*. reported a succession of various methods for the recovery of butanol produced by *Clostridium acetobutylicum*, such as gas stripping^37^,^38^, adsorption^35^, and a vapour stripping-vapour permeation (VSVP) process^36^. When gas stripping was applied intermittently in fed-batch fermentation, 195.9 g/L acetone-butanol-ethanol or 150.5 g/L butanol was obtained, and furthermore, energy consumption and water usage was reduced by at least 90%. Two-stage gas stripping was more effective for producing high-titre butanol, and a highly concentrated product containing 420.3 g/L butanol (532.3 g/L acetone-butanol-ethanol) can be obtained with this strategy. At the same time, this purification process consumed much less energy. Compared to pervaporation and gas stripping, the VSVP process produced a condensate containing 212.0–232.0 g/L butanol from a fermentation broth containing ~10 g/L butanol, which suggests that the VSVP process has great potential for efficient butanol recovery. In acetone-butanol-ethanol fermentation, the *in situ* product recovery process with activated carbon was also carried out. The results indicated that immobilized-cell fermentation with adsorption produced 54.6 g/L butanol with a productivity of 0.45 g/L·h, and a condensate containing approximately 167 g/L butanol was obtained after thermal desorption. Furthermore, this liquid phase *adsorption using* activated carbon was energy efficient and can be easily applied in butanol fermentation. In the future, integrated downstream processing technologies for fermentative 2,3-butanediol are especially required regarding yield, purity, and energy consumption.

## Materials and Methods

### Bacterial strains, plasmids, primers and reagents

The bacterial strains, plasmids and primers used in this study are listed in Table 1. All *E. coli* and *K. pneumoniae* strains were grown in Luria-Bertani (LB) growth medium (0.5% yeast extract, 1% tryptone, and 1% NaCl) at 37 °C and 30 °C, respectively. Antibiotics were added in the following amounts (per mL) when necessary: 100 μg ampicillin and 50 μg kanamycin for *E. coli* and *K. pneumoniae*, respectively. All enzymes, DNA and protein markers, and Kits were from TaKaRa Biotech (Dalian, China). Other chemicals were analytical reagent grade. All the oligonucleotide primers were synthesized in Bioasia Biotech (Shanghai, China).

### Quenching the QS system in *K. pneumoniae*

The QS mechanism of *K. pneumoniae* through the *LuxS*/*AI-2 signalling system* has played an important role in understanding the functions of QS systems. Therefore, the regulation of the QS system was determined by constructing a QS *luxS* knockout mutant strain. A 260-bp fragment from the *luxS* gene encoding a key enzyme for AI-2 synthesis was amplified from the genomic DNA of *K. pneumoniae* CICC 10018 using a PCR technique with the primers luxS-1 and luxS-2. Commercial sequencing was used to verify these mutants. The *luxS* fragment and the pUTKm plasmid were double digested with *Kpn* I and *Sca* I and then ligated between the *Kpn* I and *Sca* I sites in the plasmids. Successful ligation resulted in generating a marker-exchange plasmid, pUTKm-*luxS*, which was transformed into *E. coli* cc118 competent cells. The recombinant pUTKm-*luxS* cells were selected and confirmed using DNA sequencing and double enzyme digestion. Subsequently, the suicide vector pUTKm-*luxS* was transformed into *K. pneumoniae* CICC 10018 competent cells by electroporation. Mutants were selected on NB medium containing 800 μg/mL ampicillin and 200 μg/mL kanamycin. For each strain with a kanamycin-resistance gene inserted into the chromosome, the disruption of the locus was confirmed by PCR analysis using the primers Kan-1 and Kan-2 complementary to the KnR cassette. This mutant was named *K. pneumoniae-6*.

### Gene expression and metabolic flux analysis

The *budC* gene encodes BDH, which is involved in the biosynthetic pathway of 2,3-butanediol. An RT-PCR analysis was performed with SYBR Green technology to confirm changes in the transcription level of the *budC* gene. To prepare an external plasmid standard curve, the plasmid pGM-T-*budC* was constructed. The *budC* gene was amplified from *K. pneumoniae* with the primers budC-1 and budC-2 using genomic DNA as a template and was cloned into vector pGM-T to generate the standard plasmid pGM-T. The purified plasmid pGM-T-*budC* was serially diluted (1:10) over the appropriate concentration range (usually 10^5^–10^9^). To achieve a reliable standard curve for each measured parameter, the plasmid was PCR-amplified in five replicates for each standard dilution point over the complete standard curve range. Figure 4A shows the standard curves for the *budC* gene, which were used for the determination of *budC* gene transcription. After preparing the standard, total RNA was isolated from the *K. pneumoniae-6* sample during growth in the fermentation medium and was reverse transcribed. The resulting cDNA was directly subjected to real-time PCR with the primers F-budC and R-budC. At the same time, the production of the primary metabolites (acetic acid, ethanol, acetoin and 2,3-butanediol) by *K. pneumoniae* was assessed with the corresponding methods listed in the “Analytical methods” section.

### Batch fermentation at bioreactor scale

Submerged fermentation experiments were carried out in a bioreactor to investigate the changes in metabolic flux in *K. pneumoniae*. The fermentation medium was composed of 90 g/L glucose, 13.6 g/L KH_2_PO_4_, 5 g/L (NH_4_)_2_SO_4_, 4 g/L MgSO_4_·7H_2_O, 4 g/L Citric acid, 15 g/L yeast extract, 4 g/L NaCl, 0.4 g/L CaCl_2_, 0.08 g/L FeSO_4_ and 0.3 mL of trace elements prepared as described. The trace elements consisted of 34.2 g of ZnCl_2_, 2.7 g of FeCl_3_·6H_2_O, 10 g of MnCl_2_·4H_2_O, 0.85 g of CuCl_2_·2H_2_O, 23.8 g of CoCl_2_·6H_2_O, 0.31 g of H_3_BO_3_ and 0.25 g of Na_2_MoO_4_·2H_2_O in 1 L of deionized water. Unless otherwise specified, the submerged cultures of *K. pneumoniae* for the production of 2,3-butanediol were maintained under the following culture conditions: temperature, 30 °C; aeration rate, 4 vvm; initial pH, 7.0; and a working volume of 3.5 L. All experiments were performed in triplicate.

### Analytical methods

The primary components, ethanol, acetic acid, acetoin, and 2,3-butanediol, and other metabolites in the fermentation broth of *K. pneumonia* were detected using gas chromatography as summarized in the following steps: (1) An Agilent7890A DB-TPH column was used; (2) the detector temperature was set at 250 °C; (3) the column temperature was set at 100 °C for 2 min; (4) the temperature was increased to 180 °C at 20 °C/min and maintained for 1 min, then (5) the temperature was increased to 220 °C at 30 °C/min and was maintained for 2 min; finally, (6) the sample was injected in a volume of 1 μL.

#### Determination of enzymatic activity

1. Preparation of crude enzymeThirty millilitres of bacterial suspension collected at the indicated time points during fermentation was centrifuged at 8000 rpm for 5 min at 4 °C. The pellet was re-suspended twice with PBS and centrifuged at 8000 rpm for 5 min at 4 °C. Subsequently, 6 mL of buffer was added, and the mixture was ultrasonicated 99 × for 2s with 5-s intervals. The sonication procedure was repeated three times, followed by centrifugation at 8000 rpm for 20 min at 4 °C. The supernatant was the crude enzyme.
2. Determination of α-ALDC activityThe amount of enzyme required to convert α-acetolactic acid into 1 μmol of acetoin at 37 °C within a unit interval (min) is defined as a unit of enzymatic activity. The procedure was as follows: 25 μL of α-acetoxy-α-methyl-ethyl acetate was sufficiently mixed with 750 μL of 1 M NaOH and 750 μL of deionized water and incubated at room temperature for 20 min. The volume was then adjusted to 10 mL with PBS (pH 6). The pH was adjusted to pH 6 with 0.5 M HCl, and the volume was adjusted to 12.5 mL with PBS (pH 6). Two hundred microlitres of the final solution was mixed with 200 μL of crude enzyme and 100 μL of PBS (pH 6, containing 0.05% (w/v) Tween 80 and 600 mM NaCl). After sufficient mixing, the mixture was incubated in a 37 °C water bath for 20 min. Afterwards, 4.5 mL of a chromogenic agent (2.5 g of α-naphthol and 0.25 g of creatine in a volume of 250 mL of 1 M NaOH) was added to the mixture, which was then incubated for 40 min at room temperature, followed by OD measurement at 522 nm.
3. Determination of BDH activityThe sample solution contained 2330 μL of PBS buffer (1 mmol of ZnSO_4_, 20 μL of crude enzyme and 300 μL of 10 mM NAD^+^). This solution was the same as the control solution. Following the baseline determination, 30 μL of 1 mM 2,3-butanediol solution was added to the sample solution, while 300 μL of deionized water was added to the blank control solution. After mixing, the change in OD was measured at 340 nm.
4. Determination of DR activityThe sample solution contained 2330 μL of PBS buffer, 20 μL of crude enzyme and 300 μL of 10 mM diacetyl solution. To balance the baseline, 300 μL of 10 mM NADH solution was added to the sample solution, while 300 μL of deionized water was added to the blank control solution. After mixing the solutions, OD measurements were performed at 340 nm.

## Additional Information

**How to cite this article**: Sun, S. *et al*. The metabolic flux regulation of *Klebsiella pneumoniae* based on quorum sensing system. *Sci. Rep.* **6**, 38725; doi: 10.1038/srep38725 (2016).

**Publisher's note:** Springer Nature remains neutral with regard to jurisdictional claims in published maps and institutional affiliations.

## Figures and Tables

**Table 1:** Bacterial strains, plasmids and primers used in this study.

Strain, plasmid or primers	Relevant genotype and/or characteristics/Sequence	Source
*K. pneumoniae* CICC 10018	Wild-type strain	China Center of Industrial Culture Collection
*K. pneumoniae*-6	*K. pneumoniae* CICC 10018 *luxS* mutant, Km^r^	This study
*E. coli* DH5α	F^−^, ø80d*lacZ*ΔM15, Δ(*lacZYA-argF*)U169, *deo*R, *recA*1, *endA*1, *hsdR*17(rK^−^, mK^+^), *phoA, supE*44, λ–, *thi*-1, *gyrA*96, *relA*1	Clontech, Heidelberg, Germany
*E. coli* cc118	Km^r^, Mini Tn5 xy1AB-taltkt	Our laboratory
pUTKm	Amp^r^, Kan^r^, oriR6K, oriTRP4	Our laboratory
pUTKm-*luxS*	pUTKm containing a 0.26-kb *luxS* fragment from *K. pneumoniae* CICC 10018 inserted into the *Kpn* I-*Sca* I sites of the multiple cloning site; Km^r^	This study
pGM-T	Amp^r^, f1 ori, T7 transcription start, LacZ gene	Tiangen Biotech, Beijing
luxS-1	5′-GGGGTACCAATGGCGTGGAAATTATCG-3′	This study
	The underlined parts is the recognition site of restriction enzyme *Kpn* I	
luxS-2	5′-AAAAGTACTCAGTTCGTCGTTGCTGTTG-3′	This study
	The underlined parts is the recognition site of restriction enzyme *Sca* I	
Kna-1	5′-GAGCCATATTCAACGGGAAAC-3′	This study
Kna-2	5′-ATCGAGCATCAAATGAAACTGC-3′	This study
budC-1	5′-TCGCACTTGTTACCGGCTC-3′	This study
budC-2	5′-AAACACCATCCCGCCGTC-3′	This study
F-budC	5′-GGCGGTGTACAGCTCAAGTAAA-3′	This study
R-budC	5′-GTCAATCTCCGCCCACATC-3′	This study
